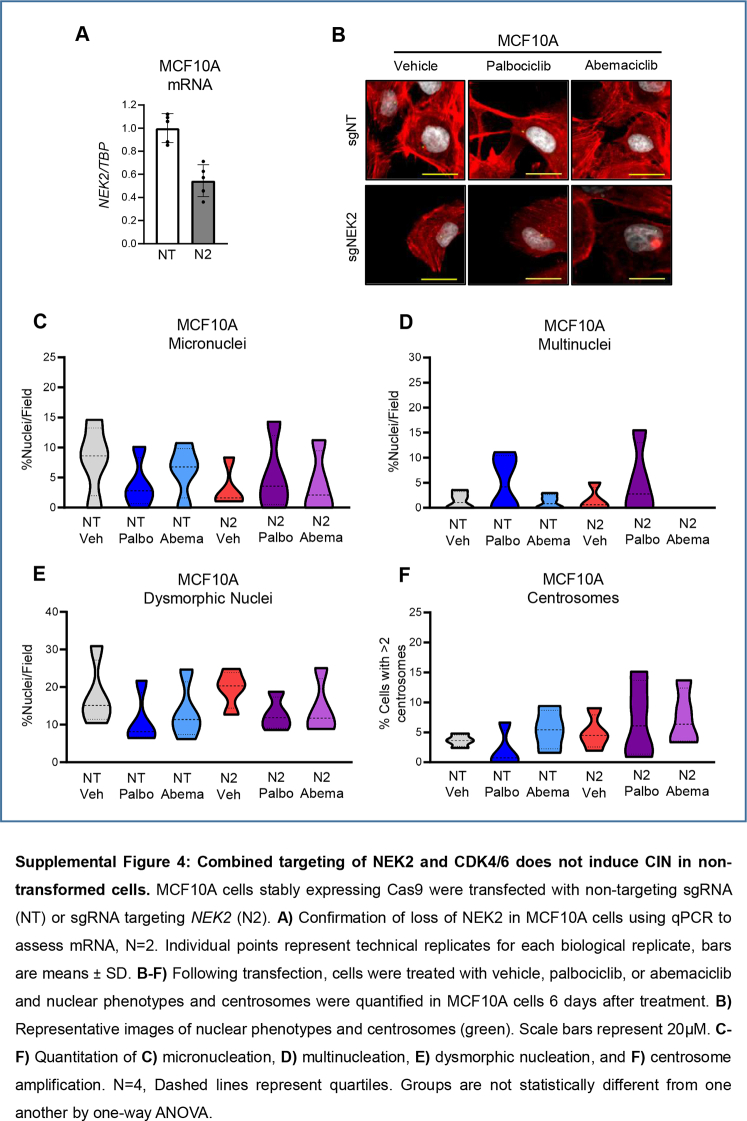# Targeting the mitotic kinase NEK2 enhances CDK4/6 inhibitor efficacy by potentiating genome instability

**DOI:** 10.1016/j.jbc.2025.108196

**Published:** 2025-01-16

**Authors:** Jessica R. Bobbitt, Leslie Cuellar-Vite, Kristen L. Weber-Bonk, Marlee R. Yancey, Parth R. Majmudar, Ruth A. Keri

**Affiliations:** 1 Department of Pathology School of Medicine, Case Western Reserve University, Cleveland, Ohio, USA; 2 Department of Cancer Biology, Lerner Research Institute, Cleveland Clinic, Cleveland, Ohio, USA; 3 Case Comprehensive Cancer Center, Case Western Reserve University, Cleveland, Ohio, USA; 4 Department of Pharmacology, School of Medicine, Case Western Reserve University, Cleveland, Ohio, USA

**Keywords:** breast cancer, CDK4/6 inhibitors, NEK2, combination therapy, mitotic catastrophe, cell cycle, cell death, mitosis, genomic instability

## Abstract

Selective inhibitors that target cyclin-dependent kinases 4 and 6 (CDK4/6i) are approved by the U.S. Food and Drug Administration (FDA) for treatment of a subset of breast cancers and are being evaluated in numerous clinical trials for other cancers. Despite this advance, a subset of tumors are intrinsically resistant to these drugs and acquired resistance is nearly inevitable. Recent mechanistic evidence suggests that in addition to stalling the cell cycle, the antitumor effects of CDK4/6i involve the induction of chromosomal instability (CIN). Here, we exploit this mechanism by combining CDK4/6i with other instability-promoting agents to induce maladaptive CIN and irreversible cell fates. Specifically, dual targeting of CDK4/6 and the mitotic kinase NEK2 *in vitro* drives centrosome amplification and the accumulation of CIN that induces catastrophic mitoses, cell cycle exit, and cell death. Dual targeting also induces CIN *in vivo* and significantly decreases mouse tumor volume to a greater extent than either drug alone, without inducing overt toxicity. Importantly, we provide evidence that breast cancer cells are selectively dependent on NEK2, but nontransformed cells are not, in contrast with other mitotic kinases that are commonly essential in all cell types. These findings implicate NEK2 as a potential therapeutic target for breast cancer that could circumvent the dose-limiting toxicities that are commonly observed when blocking other mitotic kinases. Moreover, these data suggest that NEK2 inhibitors could be used to sensitize tumors to FDA-approved CDK4/6i for the treatment of breast cancers, improving their efficacy and providing a foundation for expanding the patient population that could benefit from CDK4/6i.

A hallmark of cancer is uncontrolled proliferation and inhibitors of cell cycle progression are a mainstay therapeutic strategy. Cyclin-dependent kinases 4 and 6 (CDK4/6) are among the primary proteins responsible for controlling cell cycle entry at the G1/S transition (1, 2, 3, 4) and targeting CDK4/6 is now an Food and Drug Administration (FDA)-approved strategy for the subset of breast cancers that express estrogen receptor (ER). Such inhibitors improve progression-free survival, and in some cases, overall survival from this disease (5, 6, 7, 8, 9, 10, 11, 12, 13). Despite their initial effectiveness, nearly all patients receiving these drugs will eventually experience therapeutic resistance and recurrence of their disease. Additionally, other subsets of breast cancer are intrinsically resistant to CDK4/6 inhibitors (CDK4/6i) (14). Thus, there is a major unmet need to discover combination therapies that will enhance response to CDK4/6i and improve patient outcomes.

Entry into the cell cycle begins when mitogenic signaling stimulates the expression of the CDK4/6 binding partner and activator, cyclin D. The CDK4/6–cyclin D complex phosphorylates Rb, which typically acts as a corepressor of E2F transcription factors. However, following phosphorylation, the affinity of Rb for E2F is greatly reduced, and Rb is targeted for degradation. This frees E2F to promote its transcriptional program and triggers the transition from G1 to S phase (15, 16, 17, 18, 19, 20, 21, 22). Alterations in this pathway are some of the most commonly occurring in cancer and typically lead to rapid and uncontrolled proliferation (23, 24, 25). CDK4/6i were developed to block kinase activity and promote the accumulation of Rb. There are now three FDA-approved CDK4/6i (palbociclib, abemaciclib, and ribociclib) that prevent the expression of E2F target genes and halt cancer cells in the G1 phase of the cell cycle (26, 27, 28). The cytostatic effect of these drugs is well documented (14, 29, 30); however, some studies have also reported tumor regression, which cannot be explained by the canonical cytostatic mechanism.

Most mechanistic studies examining the cell cycle effects of CDK4/6i utilize treatment paradigms that last only a few days. However, Crozier, *et al.* demonstrated that long-term exposures cause more broad-ranging effects than stalling at the G1/S transition. Using cell cycle tracking approaches, they demonstrated that a subset of cancer cells can escape from the arresting effects of CDK4/6i and progress into S phase, generating replication errors. Such errors further stimulate exiting from the cell cycle in a p53-dependent manner. However, loss of p53, which occurs in the majority of cancers, allows continued progression through the cell cycle and accumulation of mitotic errors and chromosomal instability (CIN) (31). If extensive, this genotoxic stress can induce cell death, further limiting the proliferative potential of cancer cells. While CIN is a hallmark of cancer, it can also be a vulnerability of certain cancer cells (32). Intermediate levels of CIN are considered an enabling characteristic of cancer as they fuel the heterogeneity that promotes adaptability and overall cell fitness (33). However, as CIN increases, cells reach a threshold where the DNA content is so disrupted that they can no longer successfully divide, and this triggers cell death. This suggests that the efficacy of CDK4/6i could be potentiated by inducing intolerable CIN.

Herein, we demonstrate the ability to potentiate CDK4/6i response by inducing maladaptive levels of CIN and irreversible cell fates in models of both triple negative breast cancer (TNBC) and ER+ breast cancer. This was accomplished by combining CDK4/6i with additional CIN-inducing agents, including a bromodomain and extraterminal repeat (BET) inhibitor and mitotic kinase inhibitors. As the inhibition of many mitotic kinases is highly toxic, we identified Nima-related kinase 2 (NEK2) as a potential therapeutic target that could be combined with CDK4/6i. NEK2 is necessary for the maintenance of chromosomal stability in tumor cells (34, 35, 36, 37) and has previously been reported to be overexpressed in breast cancer cells (34, 38). We found that *NEK2* is more frequently amplified and/or overexpressed in breast cancer than other mitotic kinases and that breast cancer cells are dependent on NEK2 for growth while nontransformed cells are not. Moreover, inhibiting NEK2 potentiates the response to CDK4/6i by amplifying their ability to induce CIN *in vitro* and in mouse models of breast cancer. While effective, adding a NEK2 inhibitor to CDK4/6i did not induce overt toxicity *in vivo*. These data suggest that NEK2 may provide an ideal mitotic target for potentiating the efficacy of CDK4/6i and expanding their use in TNBC by promoting CIN and inducing mitotic cell death.

## Results

### Aneuploidy correlates with CDK4/6i sensitivity and epigenetically increasing CIN sensitizes breast cancer cells to CDK4/6i

**Supplementary Figure S1:**
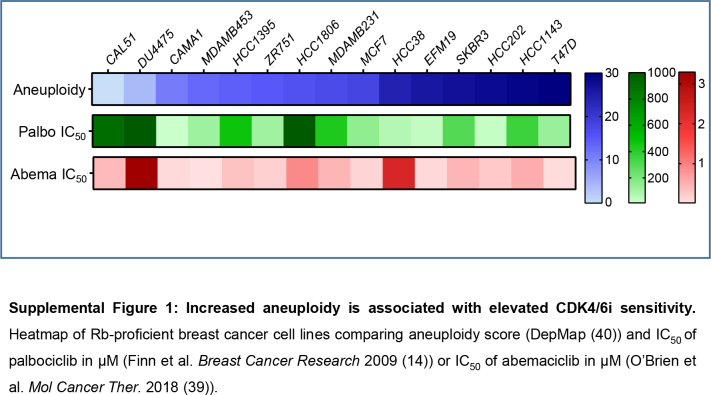


To determine if intrinsic levels of CIN may contribute to CDK4/6i efficacy, we assessed whether the effectiveness of CDK4/6i [palbociclib (14) and abemaciclib (39)] in breast cancer cell lines may be associated with their extent of aneuploidy [obtained from the broad Dependency Map (DepMap) project (40)]. For this analysis we focused on Rb-proficient cells, as loss of Rb is a common CDK4/6i resistance mechanism. While aneuploidy represents a static view of the genome, it is also indicative of ongoing CIN (41, 42). We compared the IC_50_ values for each drug with the extent of aneuploidy in each cell line and found that cell lines with high levels of aneuploidy tended to be more sensitive (*i*.*e*., have lower IC_50_ values) to palbociclib and abemaciclib (Fig. S1). This result suggests that pharmacologically increasing CIN may improve CDK4/6i responsiveness. To assess the impact of elevating CIN on CDK4/6i efficacy, we utilized two diverse cell lines with median rates of aneuploidy, MDA-MB-231, a model of TNBC, and MCF7, a model of ER+ breast cancer. Cells with median rates of aneuploidy were selected to provide the opportunity for increasing CIN over an intermediate level. Moreover, we used both TNBC and ER+ models to reveal whether increasing CIN had a generalizable effect rather than being specific to one subtype of breast cancer.

**Supplementary Figure S2:**
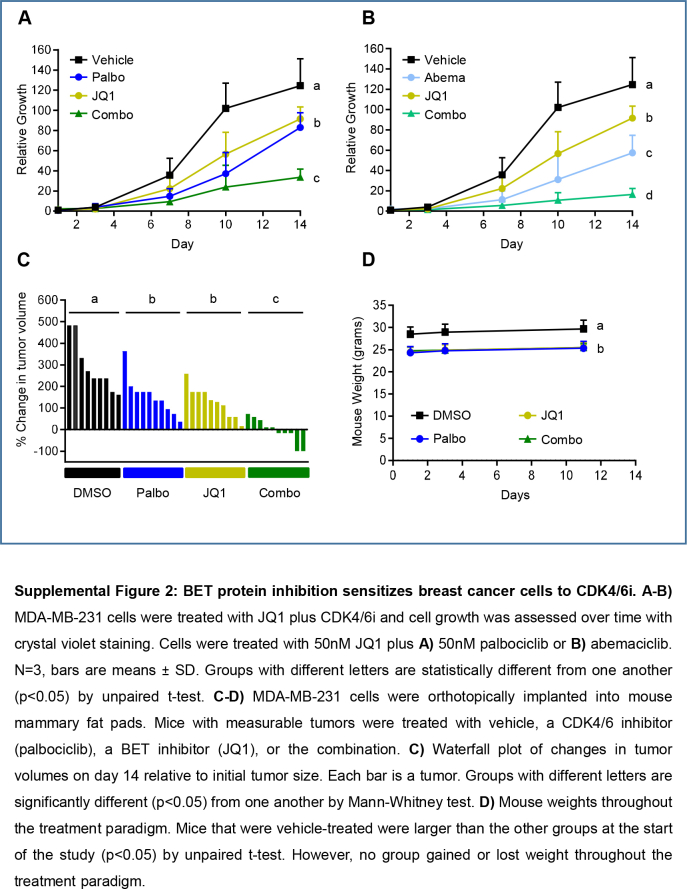


To initially evaluate the impact of combining CDK4/6i with a CIN-inducing agent, the prototypical BET inhibitor, JQ1, was utilized as a tool compound. BET proteins are required for timely progression through mitosis, and we previously reported that JQ1 induces CIN and mitosis-associated cell death in MDA-MB-231 breast cancer cells (43). Treating this cell line with low doses of JQ1 (44) led to a significant enhancement of cell growth suppression by the CDK4/6i, palbociclib, or abemaciclib, compared to single agents (Fig. S2, *A* and *B*). The impact of the combined drugs was also observed *in vivo* using orthotopic xenografts in mice, where cotreatment with JQ1 and palbociclib led to a greater suppression of tumor growth than either drug alone. Mouse weights were unchanged during the course of the experiment, indicating that the effects on tumor growth were not due to overt toxicity of the individual or combined drugs (Fig. S2, *C* and *D*). These findings are similar to those previously reported for combined efficacy of JQ1 and palbociclib, but using a different cell model (45). Together, they suggest that adding a CIN-inducing agent to CDK4/6i may increase their antitumor efficacy. While JQ1 improves tumor response to CDK4/6i, BET protein inhibitors have broad ranging effects on gene expression and this has limited their clinical development (46, 47). Thus, we sought to identify a CIN-inducing therapeutic agent that may be clinically useful while also circumventing toxicity.

### NEK2 is a potential therapeutic target for breast cancer

**Figure 1:**
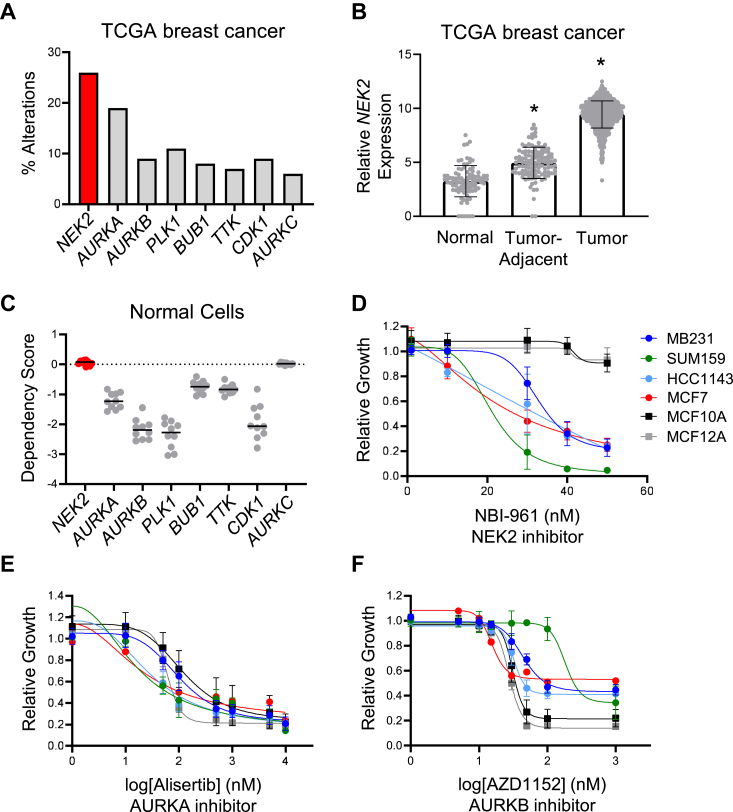
**NEK2 is a potential therapeutic target for breast cancer.***A*, alterations of select mitotic kinase genes were interrogated using data from The Cancer Genome Atlas (TCGA) for 994 breast cancers. *B*, *NEK2* expression was compared between normal (n = 92), tumor-adjacent (n = 113), and tumor tissue (n = 1119) using TCGA data for breast. Bars are means ± SD. ∗ = *p* < 0.0001 by unpaired *t* test for each group relative to normal tissue. *C*, normal, nontransformed cell lines were assessed for their dependence on select mitotic kinases utilizing data from the DepMap project. A lower dependency score suggests that the gene is more essential to those cells. *D*–*F*, dose-response curves showing the relative growth of breast cancer cells and nontransformed mammary epithelial cell lines (MCF10A and MCF12A) in response to mitotic kinase inhibitors. Each dose-response curve was repeated 2–3 times in each cell line. Cells were treated with increasing doses of the NEK2 inhibitor (*D*) NBI-961 as well as the aurora kinase A inhibitor (*E*) alisertib and the aurora kinase B inhibitor (*F*) AZD1152. Cells were treated for 7 days, and crystal violet staining was used to assess cell growth. NEK2, Nima-related kinase 2; DepMap, Dependency Map.

Mitotic kinases are essential regulators of proliferation that control genome stability by ensuring accurate segregation of chromosomes to daughter cells. As they are often overexpressed in cancer cells, mitotic kinases have received considerable focus for therapeutic development. However, the utility of drugs that target these proteins has been hampered by on-target dose-limiting toxicities that occur in rapidly proliferating nonmalignant tissues (48). Indeed, most mitotic kinases have been identified as “common essential” in the DepMap (40) as they are necessary for the growth and viability of many cell lines, including those that are nontransformed. We sought to determine if any of the mitotic kinases might be overexpressed, but not essential for normal cell viability. Analysis of data from The Cancer Genome Atlas (TCGA) revealed that compared to other common mitotic kinase genes, *NEK2* is the most frequently amplified and/or overexpressed in breast cancer (Fig. 1*A*). While deletions were included in this analysis, they occurred in <1% of tumors, and there were no tumor samples with a *NEK2* deletion. We more specifically evaluated *NEK2* expression in normal and tumor-adjacent tissues as well as breast cancers in the TCGA dataset and confirmed that *NEK2* mRNA is elevated in tumors compared to normal breast tissue (Fig. 1*B*). While *NEK2* is overexpressed in all subtypes, we previously reported that it is higher in basal and luminal B tumors than HER2 amplified or luminal A tumors (34). Moreover, high *NEK2* expression is associated with a lower probability of disease-free and relapse-free survival across all subtypes (34). Notably, unlike most other mitotic kinases, NEK2 is not required for growth of normal-like cells that were evaluated in the DepMap project, according to dependency scores (Fig. 1*C*). We also found that AURKC is not a “common essential” gene; however, its lower incidence of amplification/overexpression in breast cancer (Fig. 1*A*) reduced its therapeutic potential for inducing CIN in a large percentage of tumors.

To determine if targeting NEK2 may be selective for cancer *versus* normal cells, we treated several breast cancer cell lines (MDA-MB-231, SUM159, HCC1143, and MCF7) as well as nontransformed mammary epithelial cells (MCF10A and MCF12A) with increasing doses of a NEK2 inhibitor [NBI-961 (49)] and assessed growth. Strikingly, the nontransformed cells did not respond to the NEK2 inhibitor at doses that suppressed growth of the breast cancer cell lines (Fig. 1*D*). In contrast, the same cell lines responded to inhibitors of aurora kinase A (AURKA, alisertib) or aurora kinase B (AURKB, AZD1152) with similar IC_50_s regardless of their transformation status (Fig. 1, *E* and *F*). These data, combined with the DepMap results, suggest that cancer cells may rely on NEK2 expression/activity while normal cells may not. We and others have shown that the loss of NEK2 function causes CIN in multiple cancer models (34, 37, 50, 51). Thus, NEK2 may be a useful target for increasing CIN and improving CDK4/6i in tumors without inducing excessive toxicity.

### Targeting NEK2 enhances CDK4/6i efficacy in breast cancer cells

**Figure 2:**
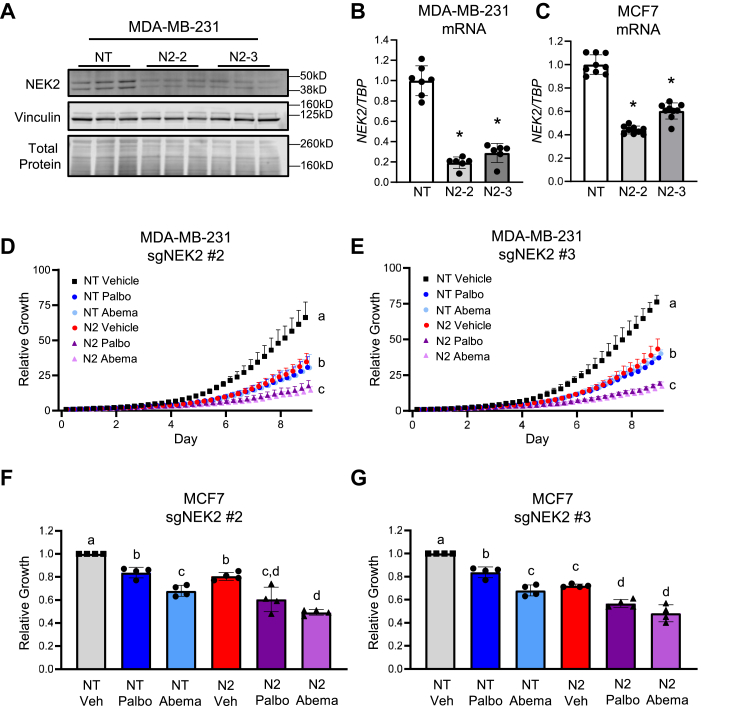
**Targeting NEK2 enhances CDK4/6i efficacy in breast cancer cells.** Cas9-expressing cells were transfected with nontargeting (NT) sgRNA or two different sgRNA-targeting *NEK2* (N2-2 and N2-3). *A* and *B*, confirmation of loss of NEK2 in MDA-MB-231 cells using (*A*) Western blot to assess protein and (*B*) qPCR to assess mRNA. *C*, confirmation of loss of *NEK2* mRNA in MCF7 cells using qPCR. Individual points represent technical replicates for each biological replicate, bars are means ± SD, ∗ = *p* < 0.0001 by unpaired *t* test compared to control. *D* and *E*, two days following transfection of MDA-MB-231 cells with two different sgRNAs targeting *NEK2* (#2 or #3), they were treated with vehicle, 50 nM palbociclib, or 50 nM abemaciclib for 9 days and relative growth was assessed using the Sartorius IncuCyte. *D*, sgNEK2 #2 and (*E*) sgNEK2 #3. N = 3, points are means ± SD. Groups with different letters are statistically different from one another (*p* < 0.05) by unpaired *t* test. *F* and *G*, two days following transfection of MCF7 cells with two different sgRNAs-targeting *NEK2*, the cells were treated with vehicle, 25 nM palbociclib, or 15 nM abemaciclib for 9 days, and relative growth was assessed by crystal violet staining (*F*) sgNEK2 #2 and (*G*) sgNEK2 #3. N = 4, individual points represent biological replicates, bars are means ± SD. Groups with different letters are statistically different from one another (*p* < 0.05) by unpaired *t* test. NEK2, Nima-related kinase 2; sgRNA, single guide RNA.

**Supplementary Figure S3:**
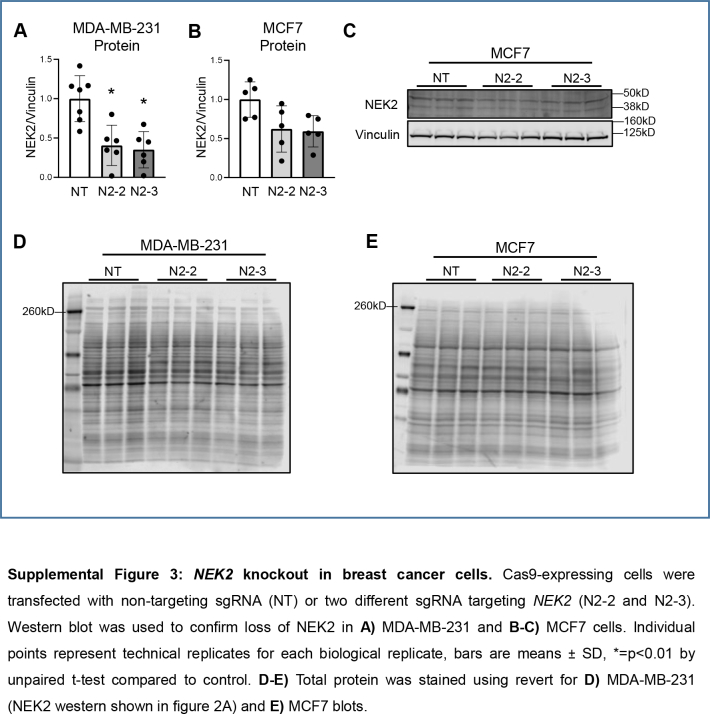


To determine if genetically targeting NEK2, which would increase CIN, could potentiate the efficacy of CDK4/6i, we used two cell lines (MDA-MB-231 and MCF7) that express NEK2 (52) but have median levels of aneuploidy (Fig. S1). NEK2 loss can inhibit the growth of breast cancer cells (34, 37, 51), precluding the development of stable, NEK2-deficient cell lines. Thus, we used a short-term KO strategy to disrupt the *NEK2* gene (53). Cells that stably express Cas9 were transfected with two different sgRNAs targeting *NEK2* (N2) or a nontargeting (NT) control. This resulted in a 40 to 80% and 40 to 60% decrease in NEK2 mRNA and protein, respectively, in these mixed populations of cells (Figs. 2, *A*–*C* and S3). The impact of NEK2 loss on the response to CDK4/6i was subsequently assessed using low doses of palbociclib or abemaciclib with treatment beginning 2 days following transfection. While NEK2 loss reduces growth on its own, it further enhances the growth suppression conveyed by CDK4/6i compared to cells transfected with the NT control (Fig. 2, *D*–*G*).

### Combined targeting of NEK2 and CDK4/6 induces CIN

**Figure 3:**
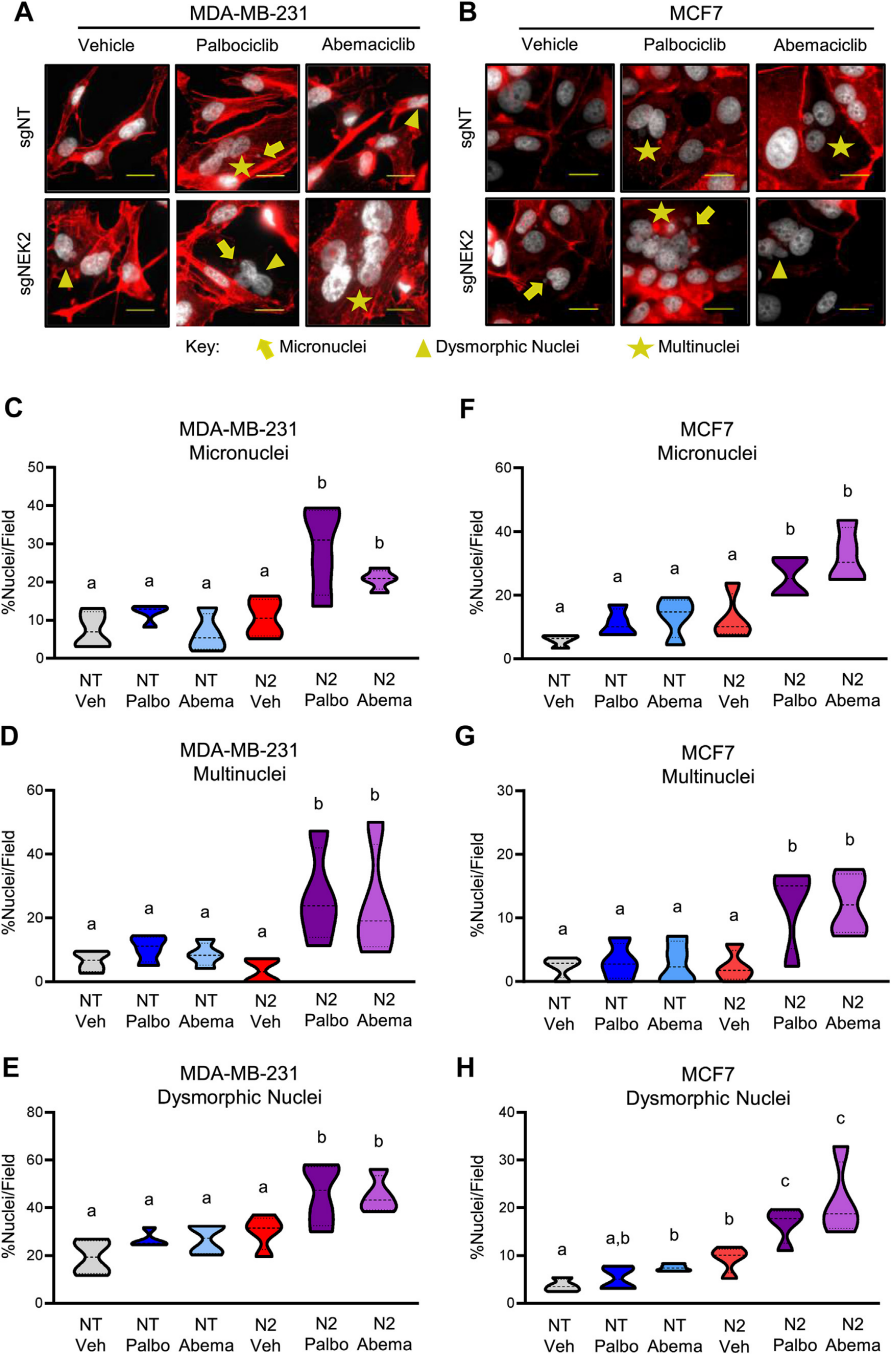
**Combined targeting of NEK2 and CDK4/6 induces CIN.** The *NEK2* gene was disrupted by transfection with sgNEK2 #3 (N2), resulting in a mixed population of cells, or cells were transfected with a nontargeting (NT) control. Following transfection, cells were treated with vehicle, palbociclib, or abemaciclib and nuclear phenotypes quantified in MDA-MB-231 cells 6 days after treatment or in MCF7 cells 8 days after treatment. *A* and *B*, representative images of nuclear phenotypes in (*A*) MDA-MB-231 and (*B*) MCF7 cells. Scale bars represent 20 μM. *C*–*E*, quantitation of (*C*) micronucleation, (*D*) multinucleation, and (*E*) dysmorphic nucleation in MDA-MB-231 cells. *F*–*H*, quantitation of (*F*) micronucleation, (*G*) multinucleation, and (*H*) dysmorphic nucleation in MCF7 cells. N = 3, *dashed lines* represent quartiles. Groups with different letters are statistically different from one another (*p* < 0.05) by unpaired *t* test. NEK2, Nima-related kinase 2; CIN, chromosomal instability.

We postulated that the improved growth suppression observed with the combination of NEK2 suppression with CDK4/6i would be associated with increased levels of CIN. To assess this directly, cells with nuclear abnormalities including multiple nuclei, micronuclei, and dysmorphic nuclei were quantified. *NEK2* gene disruption or CDK4/6i treatment alone leads to an upward trend in these nuclear phenotypes, but in most cases, these were not significantly different from controls. In contrast, the combined targeting of *NEK2* and CDK4/6 resulted in a significant increase in nuclear defects compared to vehicle or targeting either protein alone (Fig. 3, *A*–*H*). These phenotypes only occur after 6 days of treatment in MDA-MB-231 and 8 days of treatment in the slower growing MCF7 cells. Notably, the nontransformed MCF10A cells did not accumulate these CIN phenotypes in response to combined NEK2 suppression and 6 days of CDK4/6i treatment (Fig. S4). Thus, for the cells with baseline levels of aneuploidy, these defects accumulate over several cell divisions, as would be expected for the progressive accumulation of CIN that has previously been reported for CDK4/6i (31). However, these defects do not accumulate within this time frame for the more genomically stable, nontransformed cells.

### Combined targeting of NEK2 and CDK4/6 induces centrosome amplification and catastrophic mitoses

**Figure 4:**
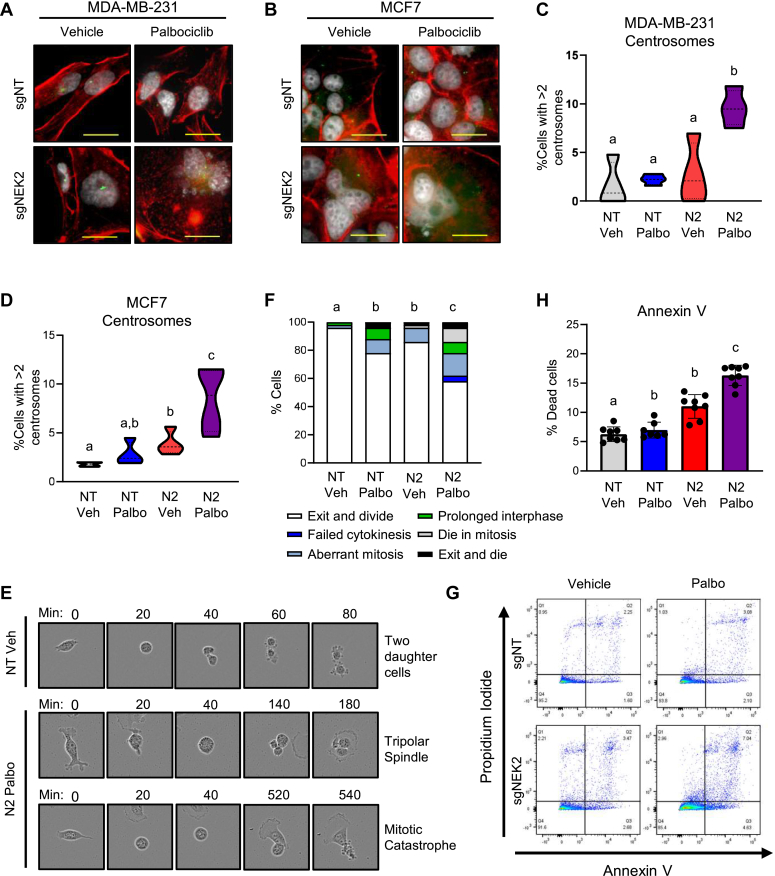
**Combined targeting of NEK2 and CDK4/6 drives centrosome amplification and catastrophic mitoses.** The *NEK2* gene was disrupted by transfection with sgNEK2 #3 (N2), resulting in a mixed population of cells, or cells were transfected with a nontargeting (NT) control. Following transfection, cells were treated with vehicle or palbociclib. *A*–*D*, centrosome numbers were quantified in MDA-MB-231 cells 6 days after treatment or in MCF7 cells 8 days after treatment. *A* and *B*, representative images of centrosomes (*green*) in (*A*) MDA-MB-231 and (*B*) MCF7 cells. Scale bars represent 20 μM. *C* and *D*, quantitation of centrosome amplification in (*C*) MDA-MB-231 (*D*) MCF7 cells. N = 3, *dashed lines* represent quartiles. Groups with different letters are statistically different from one another (*p* < 0.05) by unpaired *t* test. *E*–*H*, *NEK2* was knocked out by transfection in MDA-MB-231 cells with sgNEK2 #3 (N2). The cells were then treated with vehicle or 50 nM palbociclib. *E* and *F*, at 8–10 days following treatment, live cells were imaged with an IncuCyte microscope. Mitotic figures were identified, and their outcomes were counted for each condition. *E*, representative images and (*F*) quantitation of mitotic outcomes. N = 50 cells per condition. Groups with different letters are statistically different (*p* < 0.05) using a chi-squared test. *G* and *H*, annexin V staining and flow cytometry were used to assess cell death. *G*, representative images of flow plots and (*H*) quantification of cell death. N = 3; individual points represent technical replicates for each biological replicate, bars are means ± SD. Groups with different letters are statistically different from one another (*p* < 0.05) by unpaired *t* test. NEK2, Nima-related kinase 2.

Centrosome amplification has been reported to occur in response to *NEK2* modulation and can directly contribute to the accumulation of nuclear phenotypes (34, 54, 55, 56). Phosphorylation by NEK2 is required for separation of duplicated centrosomes and establishment of the mitotic spindle (36, 57, 58), and both its overexpression and depletion can drive centrosome amplification (36, 59, 60, 61). To determine if adding CDK4/6i potentiates the centrosome defects observed with NEK2 suppression, we quantified the proportion of cells with supernumerary centrosomes (*i.e.*, >2/cell) in cells treated individually or with the combination. Suppressing NEK2 levels alone significantly increased the percentage of cells with excessive centrosomes in MCF7 cells and modestly increased the centrosome abnormalities in MDA-MB-231 cells. In contrast, treatment with the CDK4/6i, palbociclib, alone did not have any impact on centrosome number. Most importantly, combined suppression of NEK2 and CDK4/6 drove a statistically significant increase in the percentage of MDA-MB-231 and MCF7 cells with greater than two centrosomes (Fig. 4, *A*–*D*). Notably, this combination did not drive a change in centrosome number in the nontransformed MCF10A cells (Fig. S4), suggesting that the effect of the combination on the centrosome cycle may be cancer-specific. Centrosome amplification leads to CIN and nuclear atypia, thus nuclear phenotypes observed above (Fig. 3) are likely the result of centrosome defects.

Nuclear phenotypes such as those detected with combined NEK2 suppression and CDK4/6i treatment can lead to defective mitoses (62). To determine if suppressing NEK2 in combination with CDK4/6i impacts mitotic progression and outcomes, we used live-cell imaging to track MDA-MB-231 cells as they underwent mitosis after the observation of altered nuclei (8–10 days). As they enter mitosis, cells lose their attachments and round up, exposing a visible metaphase plate. Following cell division, the daughter cells will regain attachments and ultimately, divide again. The combined suppression of NEK2 and CDK4/6 resulted in a significant increase in aberrant mitotic outcomes compared with individual treatments (Fig. 4, *E* and *F*). These include cells that divide into three daughter cells (tripolar spindle); those that complete a seemingly normal mitosis, but the daughter cells fail to subsequently divide (prolonged interphase); or cells that form a metaphase plate and then reattach without undergoing cell division (failed cytokinesis). Other cells completed division and died shortly thereafter (exit and die) or died during mitosis (mitotic catastrophe) (Fig. 4, *E* and *F*). While a low dose of palbociclib alone and *NEK2* gene disruption individually caused a statistically significant increase in these aberrant mitotic outcomes, these defects are even greater in the combination treated group (Fig. 4*F*). Together, these data demonstrate that dual targeting of NEK2 and CDK4/6 disrupts the ability of breast cancer cells to successfully complete mitosis.

Mitosis tracking revealed an increased proportion of cells undergoing mitotic catastrophe and dying during mitosis, or shortly thereafter. This is an oncosuppressive mechanism that prevents cancer cells from proliferating, typically resulting in permanent cell fates, including cell death (63). To assess the extent of cell death caused by dual suppression of NEK2 and CDK4/6, we used flow cytometry to quantify annexin V positivity. As expected, CDK4/6i treatment failed to induce a significant increase in cell death, while *NEK2* disruption increased death by ∼2-fold. More importantly, combining the *NEK2* knockout with CDK4/6i drives a further significant increase in annexin V positivity as early as day 6 (Fig. 4, *G* and *H*). These data indicate that targeting NEK2 redirects the CDK4/6i cellular response from cytostatic to cytotoxic.

### Combined pharmacological inhibition of NEK2 and CDK4/6 blocks tumor growth

**Figure 5:**
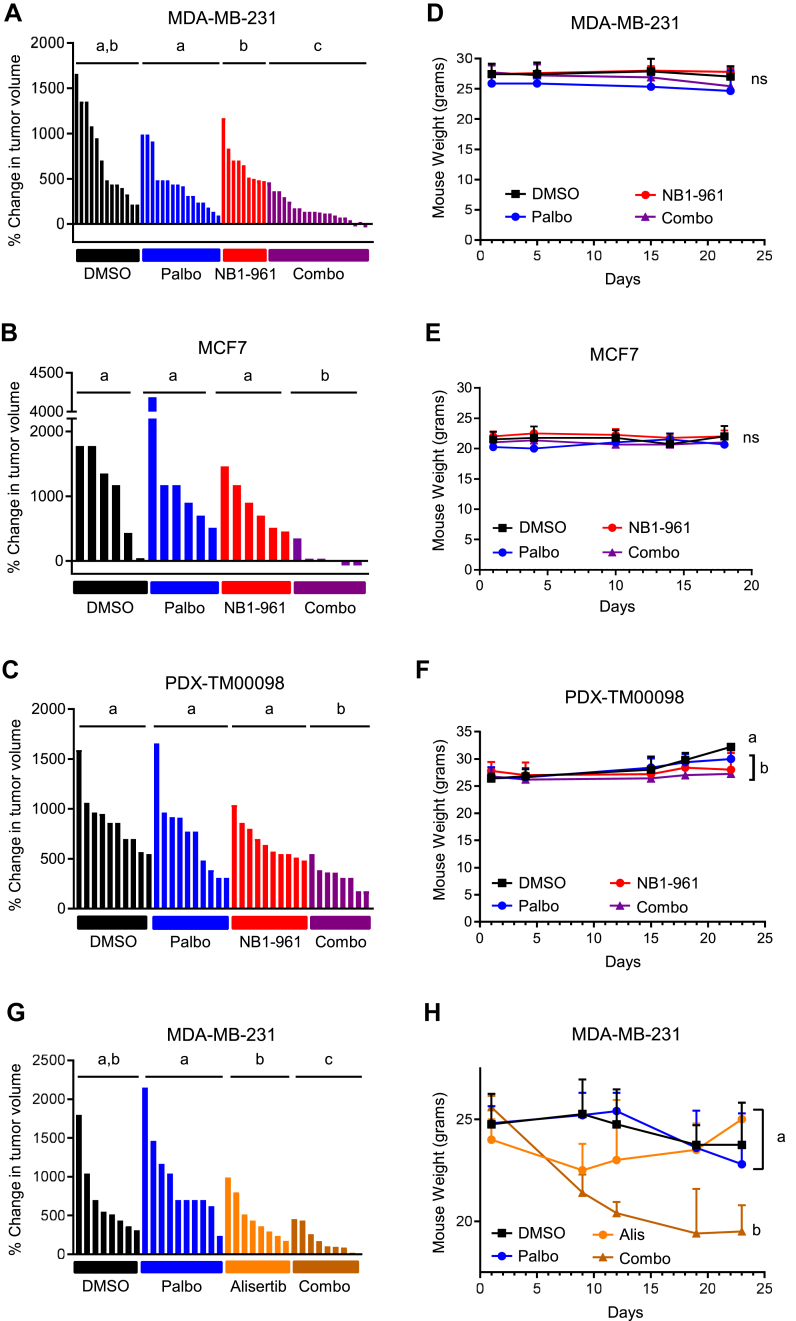
**Combined pharmacological inhibition of NEK2 and CDK4/6 suppresses tumor growth without inducing toxicity.***A*–*F*, MDA-MB-231 (*A*, *D*) and MCF7 (*B*, *E*) cells, and patient-derived xenograft (PDX) TM00098 tissue pieces (*C*, *F*) were orthotopically implanted into mouse mammary fat pads. Mice with measurable tumors were treated with vehicle, palbociclib (CDK4/6i), NBI-961 (NEK2i), or the combination. *A*–*C*, waterfall plots of changes in tumor volumes comparing the last day of treatment to initial tumor size. Each bar is a tumor. Groups with different letters are statistically different from one another (*p* < 0.05) by Mann–Whitney test. *D*–*F*, mouse weights through the duration of treatment for each experiment. Groups with different letters are statistically different from one another (*p* < 0.05) by unpaired *t* test. *G* and *H*, MDA-MB-231 cells were orthotopically implanted into mouse mammary fat pads. Following the formation of measurable tumors, mice were treated with vehicle, palbociclib (CDK4/6i), alisertib (AURKAi), or the combination for 23 days. *G*, waterfall plot of changes in tumor volumes comparing tumor volume from last day of treatment to initial tumor size. Each bar is a tumor. Groups with different letters are statistically different from one another (*p* < 0.05) by Mann–Whitney test. *H*, mouse weights were measured throughout the treatment paradigm. Groups with different letters are statistically different from one another (*p* < 0.05) by unpaired *t* test. NEK2, Nima-related kinase 2; NEK2i, Nima-related kinase 2 inhibitor; CDK4/6i, cyclin-dependent kinases 4 and 6 inhibitors.

An essential feature of a CDK4/6i sensitizer would be an ability to increase the efficacy of these drugs without inducing toxicity. Thus, we assessed the impact of NEK2 inhibition on CDK4/6i response, *in vivo* using the selective NEK2 inhibitor (NEK2i), NBI-961 (49), in three different breast cancer models. Orthotopic cell line xenografts were generated with MDA-MB-231 cells. After measurable tumors formed, the mice were treated with vehicle, NBI-961, palbociclib, or the combined drugs. Low doses of each drug were used to ensure an ability to detect increased efficacy of the combination. Individually, each drug had a minimal/limited effect on tumor growth. In contrast, combining the NEK2i and CDK4/6i resulted in a statistically significant reduction in tumor growth compared to either drug alone (Fig. 5*A*). Importantly, there was no change in mouse weight over time for any of the groups indicating a lack of overt toxicity (Fig. 5*D*). We further assessed the efficacy of the combination in two additional orthotopic xenograft models: an MCF7 cell line xenograft and a patient-derived xenograft (PDX, TM00098), which we have previously reported expresses NEK2 and responds to NEK2 inhibitors in combination with paclitaxel (34). Both models confirmed the results from the MDA-MB-231 xenograft, that is, while single agents were minimally effective at the doses used, the combination resulted in a significantly greater decrease in final tumor volume (Fig. 5, *B* and *C*). Moreover, no decreases in mouse weights were observed in any treatment group for the MCF7 model (Fig. 5*E*). For the PDX model, vehicle-treated mice gained weight while other groups maintained a constant weight (Fig. 5*F*). This may be due to an increase in tumor weight in this group over the course of the study. Together, these results reveal that *in vivo*, NEK2 inhibition is sufficient to safely sensitize several breast cancer tumor models to CDK4/6i treatment.

### Combined AURKA and CDK4/6 inhibition is toxic *in vivo*

Having observed the impact of NEK2i on the ability of CDK4/6i to induce CIN, we asked whether blocking other mitotic kinases may also increase the efficacy of CDK4/6i *in vivo*, without inducing toxicity. Supporting this possibility, AURKA/B or threonine tyrosine kinase (TTK) inhibition can reverse CDK4/6i resistance, *in vitro* (64). Using the MDA-MB-231 orthotopic mouse model, we treated tumor-bearing mice with vehicle, palbociclib, an AURKA inhibitor (alisertib), or the combination of palbociclib and alisertib. While the combined drugs resulted in significantly greater growth repression than either drug alone (Fig. 5*G*), we noted that this combination was not well-tolerated, and the mice lost 24% of their body weight within just 3 weeks (Fig. 5*H*). Compared to the *in vivo* experiments with NBI-961 and palbociclib, these findings indicate that NEK2 may be a superior therapeutic target that could improve the efficacy of CDK4/6i without inducing the dose-limiting toxicities associated with targeting other mitotic kinases.

### Combined NEK2 and CDK4/6 inhibition induces mitotic defects *in vivo*

**Figure 6:**
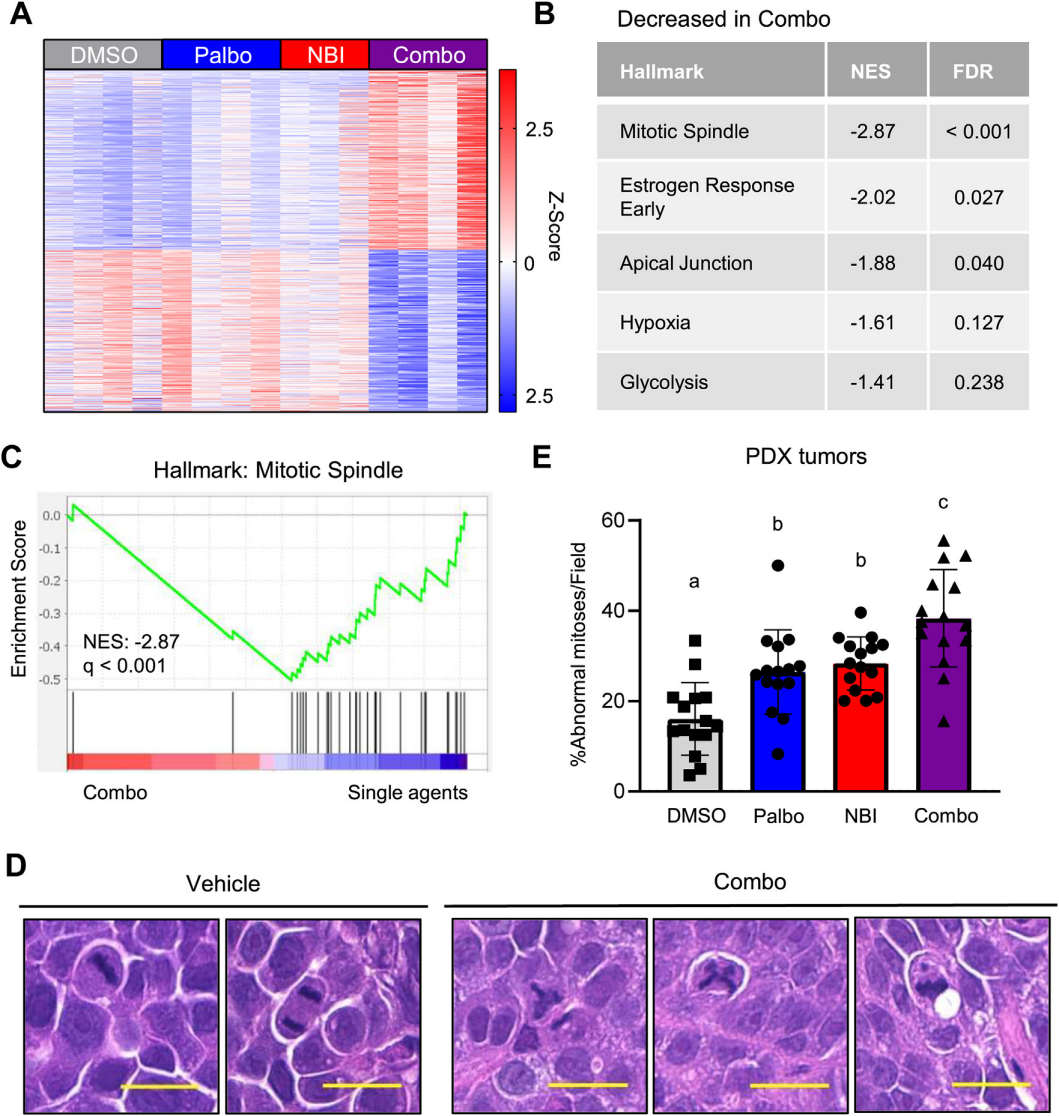
**Combined NEK2 and CDK4/6 inhibition induces mitotic defects *in vivo*.** Following the last day of treatment with palbociclib and/or NBI-961 as described in Figure 5, PDX-TM00098 tumors were harvested and processed. *A*–*C*, RNA was extracted and sent for sequencing. *A*, heat map of differentially expressed genes in the combo treated group compared to single agents, FDR <0.05. *B*, gene set enrichment analysis (GSEA) of hallmark genes reveals pathways selectively changed in tumors treated with the combination. *C*, GSEA plot of mitotic spindle genes. *D* and *E*, tumors were sectioned and stained with H&E and mitotic figures were assessed. *D*, representative images of vehicle or combo treated mitotic figures. Scale bars represent 20 μM. *E*, quantification of abnormal mitotic figures. N = 3, individual points represent technical replicates for each biological replicate, bars are means ± SD. Groups with different letters are statistically different from one another (*p* < 0.01) by unpaired *t* test. NEK2, Nima-related kinase 2; CDK4/6, cyclin-dependent kinases 4 and 6; FDR, false discovery rate; PDX, patient-derived xenograft.

To gain further insights into the mechanisms underlying the efficacy of combining NEK2 and CDK4/6 inhibition, RNA-seq was conducted using tumors collected from each treatment group from the PDX model. 1148 genes were differentially expressed in the combination treated tumors compared to those in any other treatment group (vehicle, palbociclib alone, or NBI-961 alone). The expression of 604 and 544 genes were upregulated and downregulated, respectively, in the combination compared to all other groups (Fig. 6*A*). Consistent with the *in vitro* demonstration of an elevated incidence of mitotic defects when both CDK4/6 and NEK2 were inhibited, pathway analysis revealed a decrease in the expression of mitotic spindle genes as the top differentially expressed hallmark gene set in the combination treated tumors compared to all other groups (Fig. 6, *B* and *C*). To confirm that mitotic defects occur *in vivo* in response to dual inhibition of NEK2 and CDK4/6, aberrant mitoses were directly assessed in H&E-stained tumor sections. Mitotic cells can be readily identified by their condensed chromosomes aligned at the metaphase plate, with normal cells having a clearly defined, linear orientation of chromosomal material. However, abnormal mitotic bodies where chromosomes are aligned in three or more directions (multipolar spindle) or those with chromosomes separated from the metaphase plate (micronuclei) could also be observed (Fig. 6*D*). Quantitation of the percentage of tumor cells with aberrant mitotic figures revealed that the combination of NBI-961 and palbociclib leads to a significant increase in abnormal mitoses compared to tumors treated with the individual drugs or vehicle control, *in vivo* (Fig. 6*E*). Together, these data indicate that adding a NEK2 inhibitor increases the efficacy of CDK4/6i both *in vitro* and *in vivo* by inducing mitotic defects and excessive CIN.

## Discussion

**Figure 7:**
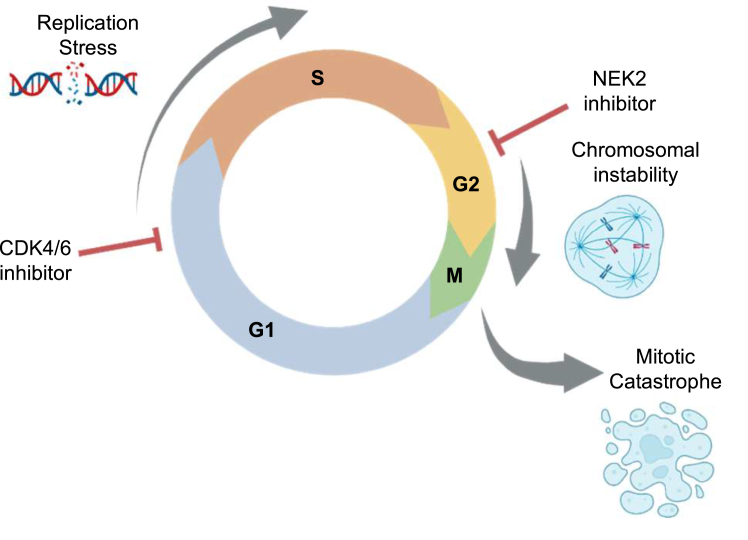
**Dual targeting of CDK4/6 and NEK2 drives an accumulation of excessive CIN, resulting in cell death.** Breast cancer cells that escape CDK4/6 inhibitor–induced G1/S cell cycle stalling can progress through S phase and accumulate replication stress (31). Targeting NEK2 then promotes the accrual of greater chromosomal defects. Over multiple divisions, the accumulation of genotoxic stress will result in programmed cell death *via* mitotic catastrophe. Figure created with BioRender.com. NEK2, Nima-related kinase 2; CDK4/6, cyclin-dependent kinases 4 and 6.

The utilization of CDK4/6i was a major paradigm shift for the treatment of breast cancers. Palbociclib, abemaciclib, and ribociclib are now used as first-line treatments for ER+/HER2− metastatic breast cancer. While originally thought to be strictly cytostatic, the unexpected finding that these drugs also drive genotoxic stress and CIN points to a path for expanding their usage through the development of rational combination therapies (31). Herein, we report that breast cancer cells can be sensitized to CDK4/6i treatment by the addition of a CIN-inducing agent. Specifically, that cotargeting CDK4/6 and the mitotic kinase NEK2 *in vitro* potentiates centrosome amplification, CIN, and mitotic errors, resulting in a significant growth defect *via* cell death (Fig. 7). Most importantly, we found that the combination of NEK2 and CDK4/6 inhibition decreases tumor volume *in vivo* in three different models without inducing overt toxicity as indicated by stable mouse weights.

Normal cells are governed by checkpoints and machinery to regulate their progression through the cell cycle and maintain their genetic content. However, cancer cells commonly lose this machinery through genetic deletions or mutations, allowing them to progress through the cell cycle unchecked. Over several divisions, they can accumulate chromosomal aberrations, initiating a chain reaction of mounting and progressive CIN. It has been estimated that 80% of solid tumors harbor CIN (65) and this has been exploited by commonly employed chemotherapies to increase CIN to levels that are incompatible with survival. For example, taxanes, which have remained a cornerstone of breast cancer treatment for half a century, exert their antitumor effects by inducing multipolar spindles leading to CIN and ultimately, cell death (66). Importantly, it has recently been reported that tumors with preexisting CIN are more sensitive to taxanes than chromosomally stable tumors, indicating that baseline levels of CIN could serve as a biomarker to identify tumors that would be particularly responsive to CIN-inducing agents (66). While CDK4/6i only modestly increase CIN, we found that their efficacy in Rb-intact breast cancer cell lines is associated with increased baseline aneuploidy, an indicator of CIN. This suggests that aneuploidy or CIN may provide a useful biomarker to predict response, a concept that requires further analysis in patients.

While effective in many tumor types, taxanes induce significant, and often permanent, side effects. The development of selective CDK4/6i for cancer was spawned by the goal of identifying cell cycle machinery that could be therapeutically targeted while also circumventing unmanageable side effects. Early studies reported that mice-deficient in both CDK4 and CDK6 undergo normal organogenesis, with most cell types developing normally. This suggested that inhibitors of both enzymes may display a broad therapeutic window (67). This led to the development and FDA approval of three CDK4/6i thus far. Similar to CDK4/6 null mice, NEK2-deficient mice are viable (International Mouse Phenotyping Consortium database) (68). Moreover, we found that a small molecule inhibitor of NEK2 was highly selective for transformed cells compared to nontransformed, normal-like mammary epithelial cell lines. Currently, we do not have a clear picture of the mechanisms underlying the dependence of the transformed cells on sustained NEK2 expression. NEK2 dependence did not correlate with *NEK2* expression levels, aneuploidy scores, rates of proliferation, or p53 status (data not shown). Thus, future studies should evaluate this dependence and may involve genetic screening to identify vulnerabilities. Regardless, the results presented herein suggest that blocking NEK2 may provide another therapeutically viable approach to target the cell cycle without inducing excessive toxicity. Moreover, it is feasible that cotargeting of CDK4/6 and NEK2 will effectively induce intolerable mitotic defects in tumor cells, while sparing normal cells. This is supported by our analyses demonstrating efficacy of the combined drugs at doses that do not induce weight loss, one surrogate of toxicity. However, dose-limiting toxicities are often driven by effects on vital organ function, which was not measured here. Therefore, future in-depth mouse phenotyping will be needed to further discover any short- or long-term untoward effects of combining these or other inhibitors targeting CDK4/6 and NEK2.

Two breast cancer cell lines were used for this study that originate from tumors of different molecular subtypes. MCF7 cells are a model of ER+, luminal breast cancer, the subtype for which CDK4/6i are FDA-approved. In contrast, MDA-MB-231 cells are a model of TNBC, which has been reported to have varying degrees of intrinsic resistance to CDK4/6i (14). Parallel experiments were also completed in the MCF10A cell line, a model of nontransformed mammary epithelial cells. It is therefore particularly striking that dual targeting of NEK2 and CDK4/6 is effective at augmenting CIN and repressing cell growth in both models of breast cancer. Moreover, the PDX used in this study is also a model of TNBC, further supporting the efficacy of the combination in this breast cancer subtype. Luminal and TNBC subtypes of breast cancer are highly divergent and have several molecular differences that could contribute to distinct responses to CDK4/6i, including RB status, p53 status, and differential expression of G1 proteins (14, 69, 70, 71, 72). For example, *RB1* mutations, which have been described as negative biomarkers of response to CDK4/6i, are much more common in TNBC (71, 73, 74, 75). In contrast to these differences, we note that the MDA-MB-231 and MCF7 models have similar baseline levels of aneuploidy, according to DepMap (40), but the MCF10A cells retain a more stable genome (76, 77). We suggest that an unstable genome may portend the likelihood of accumulating excessive CIN in response to combined inhibition of CDK4/6 and NEK2. The studies presented here provide a foundation for discovering the mechanisms driving aneuploidy and determining if these also contribute to the combined efficacy of inhibitors of these cell cycle-regulating kinases.

In addition to reports that short-term CDK4/6i drives CIN, it has also been demonstrated that models that acquire CDK4/6i resistance after prolonged exposures also display mitotic defects (64). *In vitro* studies have identified several cell cycle proteins as candidate mediators of CDK4/6i resistance, including other mitotic kinases, and inhibitors of those kinases have been shown to effectively block proliferation (64, 70). Further, analysis of breast tumor biopsies revealed that resistant tumors were associated with the high expression of G2/M genes, which includes mitotic kinases (78). The models used herein evaluated the ability of NEK2i to improve CDK4/6i efficacy prior to extended treatment and acquisition of resistance mechanisms. Whether this approach will also be effective once CDK4/6i resistance or Rb mutation occurs remains to be determined. Importantly, NEK2 has not yet been identified as a resistance mechanism using molecular profiling, suggesting that changes in the *NEK2* gene itself may not drive resistance. However, the ability of NEK2i to induce mitotic dysfunction and excessive CIN indicates that these inhibitors should act as CDK4/6i sensitizers in the context of a variety of cell cycle–associated mechanisms of resistance that may arise.

In conclusion, the data presented herein suggest that NEK2 inhibitors may provide a novel approach for increasing the efficacy of FDA-approved CDK4/6 inhibitors in the treatment of aggressive and chromosomally unstable cancers. This includes certain ER+ breast tumors for which CDK4/6i are currently used, as well as the highly aggressive TNBC subtype where CDK4/6i are currently being tested. Importantly, there are now hundreds of clinical trials taking place worldwide in several cancer types examining the impact of CDK4/6i as single agents or in combinations. The data presented herein provides a rationale for combining CDK4/6i with CIN-inducing agents, including NEK2i, and for utilizing CIN as a biomarker of response. Overall, these findings lay a foundation for further expanding the patient population that could benefit from CDK4/6i.

## Materials and methods

### Cell culture

SUM159 cells were purchased from Asterand Bioscience. All other cell lines were purchased from the American Type Culture Collection. MDA-MB-231, HCC38, and HCC1143 cells were cultured in RPMI-1640 (Corning) with 1% penicillin/streptomycin (pen/strep; Gibco) and 10% fetal bovine serum (FBS; Atlanta Biologicals). SUM159 cells were cultured in Ham’s F-12 media (Gibco) with 10 mM Hepes (Gibco), 1 mg/ml hydrocortisone (Sigma-Aldrich), 10 mg/ml insulin (Sigma-Aldrich), 1% pen/strep, and 5% FBS. MCF7 cells were cultured in Dulbecco's modified Eagle's medium (Corning), 10 mg/ml insulin, 1% pen/strep, and 10% FBS. MCF10A and MCF12A cells were cultured in Dulbecco's modified Eagle's medium/Ham’s F-12 (Corning), 10 ug/ml cholera toxin (MilliporeSigma), 1 mg/ml hydrocortisone, 10 mg/ml insulin, 20 ng/ml epidermal growth factor (Thermo Fisher Scientific), 1% pen/strep, and 5% horse serum (Invitrogen). All cell lines were maintained at 37 °C and 5% CO_2_ and were used within 10 passages of thawing. All cell lines have been validated by STR testing (LabCorp) and were continuously tested for *Mycoplasma pulmonis* and *Mycoplasma* spp using the MycoAlert PLUS *Mycoplasma* Detection Kit (Lonza).

### Gene targeting

MDA-MB-231, MCF7, and MCF10A cells that stably express Cas9 were generated using Lentiviral Cas9 Nuclease Reagents (Dharmacon) according to manufacturer’s instructions. Following transduction, cells were selected using Blasticidin (Gibco) at 20 μg/ml for MDA-MB-231, 10 μg/ml for MCF7, and 20 μg/ml for MCF10A cells. Cas9 activity was validated with Edit-R synthetic single guide RNA (sgRNA)-positive control (Lethal control #1, Dharmacon) as previously described (53). Cells stably expressing Cas9 were then transfected with sgRNA targeting the *NEK2* gene to achieve transient knockout of NEK2. Two different single guides targeting NEK2 (Dharmacon, sgNEK2 #2: SG-004090-02, sgNEK2 #3: SG-004090–03) and one NT control sgRNA (Dharmacon, sgNT: U-009501-01) were utilized in these studies. sgRNA at a final concentration of 62.5 nM was mixed with Dharmafect #4 (Horizon, T-2004) for MDA-MB-231, Dharmafect #1 (Horizon, T-2001) for MCF7, and Dharmafect #2 (Horizon, T-2002) for MCF10A at a dilution of 1:250 in pen/strep-free, serum-free media for 20 min at RT°. The mixture was added to cells and incubated for 6 h at 37 °C and 5% CO_2_ before diluting with an equal volume of pen/strep-free media. The day following transfection, cells were collected and replated for each experiment. For all experiments, cells were plated at a low density to prevent a G1 arrest due to contact inhibition. Loss of NEK2 was confirmed at the RNA and protein level by reverse transcription, quantitative polymerase chain reaction (RT-qPCR) and Western blot, respectively.

### Drug treatment and dose response curves

NBI-961 (previously “CMP3a”, MedKoo Biosciences, 407833), alisertib (MedChemExpress, HY-10971), AZD1152 (MedChemExpress, HY-10127), palbociclib (MedChemExpress, HY-A0065), and abemaciclib (MedChemExpress, HY-16297A) were resuspended in dimethyl sulfoxide (DMSO, Sigma). For single agent dose response curves, cells were plated in 96-well plates and the next day treated with DMSO or individual inhibitors (DMSO at a final concentration of 0.1%). Cell growth was assessed by crystal violet staining (0.05% w/v crystal violet, 1% of 37% formaldehyde, 1% PBS, and 1% methanol), which was diluted in 10% acetic acid and absorbance was quantified using a GloMax Explorer plate reader (Promega, GM3510). For combination studies with sgNEK2, cells were replated the day following transfection. Twenty-four hours later, they were treated with DMSO or CDK4/6i. MDA-MB-231 and MCF10A cells were treated with a final concentration of 50 nM palbociclib or 50 nM abemaciclib, and MCF7 were treated with 25 nM palbociclib or 15 nM abemaciclib.

### Cell growth assays

For JQ1 and CDK4/6i combination growth assays, MDA-MB-231 cells were plated in 24-well plates, and 24 h later treated with DMSO control, 50 nM palbociclib, 50 nM JQ1, or the combination. Live cells were counted using trypan blue exclusion and a Countess II Fl (Thermo Fisher Scientific) for up to 14 days. For sgNEK2 and CDK4/6i combination growth assays, Cas9-expressing MDA-MB-231 and MCF7 cells were plated, and the following day transfected with NT or *NEK2*-targeting sgRNAs as described above. The following day, cells were replated into 24-well plates. Twenty-four hours later cells were treated with DMSO control, palbociclib (50 nM for MDA-MB-231 and 25 nM for MCF7), or abemaciclib (50 nM for MDA-MB-231 and 15 nM for MCF7) for 9 days. For MCF7 cells, growth was assessed by crystal violet staining as described above. For MDA-MB-231 cells, growth was assessed over time utilizing live-cell imaging with the IncuCyte S3/SX1 Live-Cell Analysis System (Sartorius). Nine images per well were collected every 6 h, and confluency was tracked over time utilizing the Live-Cell Analysis Software (https://www.sartorius.com/en/products/live-cell-imaging-analysis/live-cell-analysis-software) by Sartorius.

### Western blotting

Cells were lysed using NP-40 (Millipore Sigma, 18896) assay buffer containing protease (MilliporeSigma, 539138) and phosphatase (MilliporeSigma, 4906825001) inhibitors. Cell lysate was incubated on ice for 30 min with intermittent mixing and spun at 10,000 RPM for 10 min at 4 °C. Supernatant was collected, and protein was quantified by Bio-Rad Protein Assay (Bio-Rad, 5000001). Samples were diluted with an equal volume of 2x Laemmli buffer (Bio-Rad, 1610737) plus 5% 2-mercaptoethanol, boiled, and loaded into precast polyacrylamide gels (Novex 4–20% Tris-Glycine Mini Gels, Thermo Fisher Scientific, XP00100BOX) with molecular weight markers (LI-COR, 928-60000). Protein was transferred from the gels to Immobilon-FL PVDF membrane (Millipore Sigma, IPFL00010) at 100 milliamps for 16 h. Total protein was stained with REVERT (LI-COR, 926-11021). Membranes were blocked with 5% bovine serum albumin plus 20% SDS in Tris-buffered saline with 0.05% Tween-20 (TBST). The following primary antibodies were utilized: anti-Vinculin (Sigma-Aldrich, V9131, 1:5000), and anti-NEK2 (BD Biosciences, 610594, 1:500). Primary antibodies were diluted in 5% bovine serum albumin-TBST and membranes were incubated for 3 days at 4 °C. Membranes were washed in TBST and incubated in secondary antibody (LI-COR, 926-32210, 1:5000 for NEK2 and 1:20,000 for Vinculin) for 1 h in the dark at room temperature. Blots were imaged using the LI-COR Odyssey Fc. Densitometry and protein levels were normalized relative to Vinculin. Antibodies were validated using siRNA and blotting for the targeted protein.

### RNA/qPCR

RNA was isolated from cells with TRIzol reagent (Ambion, 155596016). RNA was treated with a DNA-free DNAse kit (Ambion, AM1906) per manufacturer’s instructions and its concentration was measured using a NanoDrop One (Thermo Fisher Scientific, 13-400-509). RNA quality was confirmed using a 1.5% agarose gel and the 28S/18S bands were examined. Complementary DNA was generated using Superscript IV reverse transcriptase (Thermo Fisher Scientific, 18090010) with random primers (Thermo Fisher Scientific, 48190011) following the manufacturer’s protocol. Quantitative real-time PCR was performed on a StepOnePlus Real-Time PCR System (Thermo Fisher Scientific). The following TaqMan real-time assays were utilized (Thermo Fisher Scientific; NEK2: Hs06629033_g1, TBP: Hs00427620_m1). Gene expression was normalized to TBP.

### Flow cytometry

For cell cycle analyses, cells were harvested with 0.25% trypsin, fixed in 70% ethanol for 10 min at RT°, and incubated in propidium-iodide/RNase A solution (100 μg/ml propidium iodide, 0.1% Nonidet P-40, 0.1% NaN3, and 1.2% RNase A) for 30 min at 37 °C. For annexin V analysis, cells were harvested with 0.25% trypsin, and the Dead Cell Apoptosis Kit with annexin V was used (Thermo Fisher Scientific, V13245) according to manufacturer’s protocols. Flow cytometry was performed with an LSRFortessa Flow Cytometer (BD Biosciences). Gating analysis was completed with FlowJo (https://www.flowjo.com/solutions/flowjo).

### Fluorescence imaging of nuclei

Cells were grown in 6-well plates on coverslips and fixed with 3.7% formaldehyde for 10 min, then permeabilized with 0.1% Triton X-100. To visualize cell bodies, slides were stained with Texas Red-X Phalloidin (Invitrogen, T7471) for 20 min at RT° in the dark. To visualize centrosomes, slides were incubated with a γ-tubulin antibody conjugated to Alexa Fluor 488 (Abcam, AB205475) overnight at 4 °C. Slides were counterstained and mounted with ProLong Diamond Mountant with 4′,6-diamidino-2-phenylindole (Invitrogen, P36962). Images were collected at 60x magnification using the all-in-one fluorescence microscope BZ-X800 (Keyence, Osaka). The total number of cells per field were quantified, and the proportion of cells with micronucleation, multinucleation, dysmorphic nuclei, or centrosome amplification were assessed. At least 50 cells per condition per biological replicate were evaluated.

### Live-cell imaging

The day following transfection, MDA-MB-231 cells were replated in 6-well plates. The following day cells were treated with DMSO or 50 nM palbociclib, plates were placed in an IncuCyte S3/SX1 Live-Cell Analysis System (Sartorius), and images were collected at 10x magnification every 20 min from days 8 to 10. The beginning of mitosis was determined to be the first image prior to the visualization of the metaphase plate. The end of mitosis was determined when the resultant cells readhered to the plate or died. The following cell fates were quantified: exit and divide (two daughter cells), exit and die (one or both cells die shortly following the end of mitosis), die in mitosis (after the metaphase plate forms, the cell dies), prolonged interphase (one or more cells fail to divide again following the end of mitosis), or failed cytokinesis (after mitosis begins, the cell readheres to the plate without dividing). Fifty mitotic cells per condition were quantified.

### *In vivo* studies

All *in vivo* experiments were approved by the Institutional Animal Care and Use Committee at Case Western Reserve University or at the Cleveland Clinic Lerner Research Institute. Mice were housed in microisolator units maintained on a 12-h light/dark cycle and were given standard chow and water. Tumor pieces from TM00098 (The Jackson Laboratory), or MDA-MB-231 or MCF7 cells were orthotopically implanted into both inguinal mammary fat pads of adult female NOD/scid/γ mice. Mice with palpable tumors (∼300 mm^3^) were randomized into four treatment groups: vehicle (DMSO), palbociclib alone, CIN-inducing agent alone, or the combination of palbociclib and a CIN-inducing agent, depending on the experiment. The following doses were used for treatment: palbociclib (50 mg/kg, IP injection), NBI-961 (10 mg/kg, IP), JQ1 (50 mg/kg, IP), and alisertib (20 mg/kg, PO). Mice were treated daily 5 days/week for the duration of the experiment, except in the MCF7 model where mice were treated with palbociclib only 2 days/week. Tumor size was measured by calipers twice weekly and mouse weight was measured weekly. On the last day of treatment, tumors were harvested, bisected, and one half was fixed in 4% paraformaldehyde while the other half was placed in RNAlater stabilization solution (Thermo Fisher Scientific, AM7020), frozen at −20 °C, and subsequently processed for RNA analysis.

### RNA-sequencing

Following treatment with vehicle, palbociclib, NBI-961, or the combination as described above, TM00098 PDX tumors were collected and frozen at −80 °C. For each group, total RNA was isolated from frozen tissues of the median responding tumors according to the manufacturer’s protocol (RNeasy Plus Mini Kit, Qiagen). For vehicle, palbociclib, and the combination, four tumors were assessed, and for NBI-961, three tumors were assessed. Quality control, library preparation and sequencing on the Illumina platform was completed by Novogene Corporation Inc. Paired end reads were analyzed with Partek Flow Software (https://www.illumina.com/products/by-type/informatics-products/partek-flow.html). STAR was used to align reads to Hg38 and DESeq2 was used to assess differentially expressed genes with a false discovery rate of <0.05.

### H&E staining

Following treatment with vehicle, palbociclib, NBI-961, or the combination as described above, formalin-fixed tissue from the TM00098 PDX tumors were paraffin-embedded, sectioned, and stained with H&E. Embedding and staining was performed by the imaging core at the Lerner Research Institute. H&E-stained slides were imaged by brightfield microscopy with the all-in-one fluorescence microscope BZ-X800 (Keyence, Osaka). Cells undergoing mitosis were identified per condition, and the percentage of aberrant mitotic figures was quantified. At least 50 mitotic cells were quantified per condition.

### Public data analysis

Data from TCGA was downloaded from cBioPortal to assess alterations in *NEK2, AURKA, AURKB, PLK1, BUB1, TTK, CDK1,* and *AURKC* in breast cancer. Data are from 994 patient samples, and alterations include high mRNA expression, amplification, and deep deletion. mRNA expression z-scores were compared to the tumors that were diploid for each gene, with high mRNA expression defined as a z-score >2.

Data from TCGA and GTEx was used to assess *NEK2* expression across tissue types. Bulk RNA-seq data from 1119 tumor samples (GSM1536837), 113 tumor-adjacent samples (GSM1697009), and 92 normal samples (GSE86354) was downloaded from GEO. Raw counts were normalized using DESeq2 (https://bioconductor.org/packages/release/bioc/html/DESeq2.html), and *NEK2* expression values were log2-transformed after addition of a pseudocount of 1 and plotted (79). A Student's *t* test was used to assess statistical significance between each group.

Data from the Broad Institute’s DepMap was utilized to investigate the dependency of noncancerous cells on the following mitotic kinases: *NEK2, AURKA, AURKB, PLK1, BUB1, TTK, CDK1,* and *AURKC*. The noncancerous cell lines included in this analysis are HS729 (fibroblasts), HA1E (renal epithelial cells), BPH1 (benign prostatic hyperplasia cells), ARH77 (B lymphoblast cells), IM9 (B lymphocytes), RPE1SS48, RPE1SS77, RPE1SS6, RPE1SS119, and RPE1SS51 (derivatives of RPE-1, retinal pigment epithelial cells).

### Statistical analyses

For *in vitro* analyses, statistical significance was determined using two-tailed Student’s *t* test for all experiments except mitotic catastrophe where a chi-squared test was used. For *in vivo* experiments, a Mann–Whitney U test was used to assess statistical significance. *p* values of <0.05 were considered significant. *In vitro* experiments were performed at least three independent times with intraexperimental technical replicates. The mean of the technical replicates is shown with variability represented by SD.

## Data availability

Raw data from RNA-seq were deposited in GEO, under accession number GSE277102.

## Supporting information

This article contains supporting information (14, 39).

## Conflict of interest

The authors declare that they have no conflicts of interest with the contents of this article.

## Figures and Tables

**Supplementary Figure S4:**